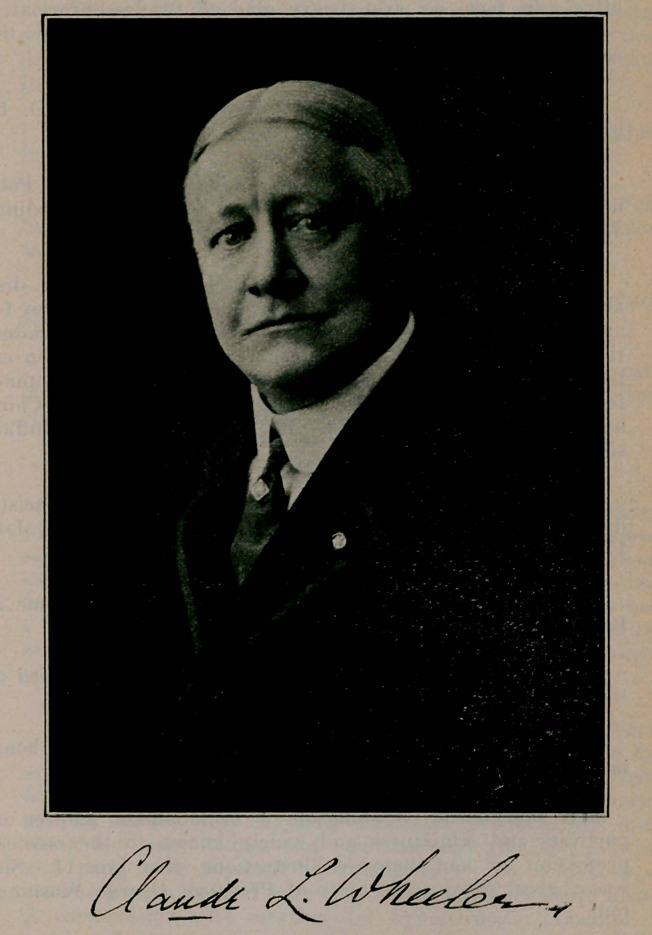# Dr. Claude Lamont Wheeler

**Published:** 1917-02

**Authors:** 


**Dr. Claude Lamont Wheeler,** McGill 1889, died at his home in Flatbush, Brooklyn, Dec. 30, of pneumonia, aged 53. He succeeded Dr. Frank P. Foster in 1909, as editor of the N. Y. Medical Journal, having previously served on the staff of American Medicine, and, since 1902, of the N. Y. Medical Journal. He was for some time a member of the staff of the Manhattan Eye, Ear, Nose and Throat Hospital.

Claude L. Wheeler

## Figures and Tables

**Figure f1:**